# Preventing the preventable: Assessing the burden of incessant caesarean deliveries in select Indian states using NFHS-5

**DOI:** 10.1371/journal.pone.0320041

**Published:** 2025-04-23

**Authors:** Punit Mishra, Retna Sheeja Pushpa Raj, Aditi Aditi, Muthusamy Sivakami

**Affiliations:** 1 PopulationCouncil Consulting, New Delhi, Delhi, India; 2 All India Institute of Medical Sciences (AIIMS), New Delhi, Delhi, India; 3 Tata Institute for Social Sciences, Mumbai, Maharashtra, India; Indian Institute of Dalit Studies (IIDS), INDIA

## Abstract

The World Health Organization (WHO) recommends that life-saving Caesarean sections (CS) should account for 10–15% of deliveries. Southern states of India have good obstetric facilities and better public health systems than other parts of India. However, CS rates in select states are considerably higher. This study examines the prevalence and determinants of preventable CS among mothers in select Indian states, specifically the states that have high institutional deliveries (>95%) viz. Andhra Pradesh, Kerala, Karnataka, and Tamil Nadu. It further compares the complications faced by CS and vaginal delivery cases. Data has been derived from a cross-sectional survey, the National Family Health Survey-5. Bivariate and Logistic regression analyses were used. The main outcome measured is Preventable C-sections, defined as per conditions in Robson’s criteria of 10-group classification based on obstetric characteristics for a woman. Results show that the preventable CS rates in these selected states were much higher than at the national level. Key correlates were higher education and middle socioeconomic status. The study highlights the need for policy reforms, maternal education, and healthcare system improvements to reduce unnecessary CS in select states of India, where the health system is exceptionally good but , have another health burden in form of demand for unnecessary caesarean deliveries.

## Introduction

Caesarean delivery (C‑section/ CS) is a surgical process of delivering a baby through a laceration in the mother’s abdomen and uterus. According to the World Health Organization (WHO), C-sections effectively save maternal and infant lives but should be done only when required for medically indicated reasons [1]. The WHO advocates a maximum range of ‘10–15%’ of C-sections at community levels to improve maternal and child healthcare outcomes substantially. Nevertheless, it has been proved that higher percentages of CS deliveries do not reduce maternal mortality rates [1]. The unnecessary C-section rates are calculated by considering the CS rates more than the acceptable range suggested by WHO. Studies report that approximately 8.8 million unnecessary CS occur globally, of which close to 6 million are in upper-middle-income countries [2]. The global surge in caesarean section (C-section) rates has sparked significant public health concerns due to its potential implications for maternal and neonatal outcomes. While C-sections are essential in managing obstetric complications, their excessive use has been associated with increased maternal morbidity, neonatal respiratory issues, and long-term health risks [3]. With at least 1.8 million unnecessary CS, India accounts for the highest number among lower-middle-income countries [3,4]. In India, this trend is particularly pronounced, with states like Telangana and Tamil Nadu reporting C-section rates exceeding 50% in private healthcare facilities, often influenced by financial incentives and non-medical factors. Addressing this overuse necessitates evidence-based interventions and policy reforms [5,6]. India has witnessed a decline in MMR from 424 per 100000 live births in the early 1990s to 93 per lakh live births in 2020 [5]. A significant 21.9% decline in MMR in India is attributable to increased access to essential maternal health services. Yet, an interesting dichotomy is observed in the case of maternal healthcare in India. While India still accounts for nearly 1/5th of the global MMR, it also has a substantially high percentage of C-section deliveries at 21.5% as per the latest NFHS report [6]. Specifically, some Indian states, namely, Tamil Nadu, Andhra Pradesh, Telangana, Karnataka, and Kerala, with more than 96.5% institutional deliveries, better health indicators, and health facilities [6,7]. There also exists a substantially higher percentage of C-section deliveries, ranging from 31.5% to 60.7% in these states [8]. The C-section rates in these states are higher than in some of the countries of Western Europe and North America [9]. While the high prevalence of CS deliveries coincides with a high percentage of institutional deliveries, an alarmingly high prevalence of C-section deliveries over and above the WHO recommendations and global averages indicates the overuse of C-section procedures in these Indian states. Hence, it may be argued that a substantial percentage of C-sections could be medically unwarranted or preventable [9]. Studies have discussed that medically unwarranted C-sections introduce avoidable complications and call for C-sections to be judiciously performed [10,11]. Researchers report that c-sections, specifically intrapartum c-sections carry a greater risk of severe acute maternal morbidity (SAMM) compared to vaginal delivery [9]. In subsequent pregnancies, women undergoing CS have greater odds of adverse outcomes such as abnormal placentation, uterine rupture, and preterm delivery [12]. Moreover, a previous history of CS is one of the primary indications for CS delivery [9], indicating that C-sections extend the health risks to subsequent pregnancies [13].

While literature agrees that unwarranted c-sections are on the rise, the decision to conduct a c-section delivery is largely based on the physician’s discretion, with or without request from the mother/family members [14]. Undeniably, medical support is essential for all deliveries; C-sections are usually warranted if the pregnancy is considered to be high-risk for the mother and/or fetus or if there is an intrapartum complication such as breech position, prolonged labor, or excessive bleeding [15]. The government of India outlines an exhaustive list of high-risk pregnancies, among which medical intervention through C-section may be warranted [14,15]. In South-Indian states with high coverage of antenatal care (ANC) and institutional deliveries, a prior decision to undertake a C-section will comprise all the possible prepartum medical indications for a necessary C-section. In this regard, a preventable C-section can be defined as a C-section delivery that was not planned prior to the onset of labor, conducted at full term, and done without any intrapartum complications such as breech presentation, transverse lie, prolonged labor, and excessive bleeding. This definition provides a conservative estimate of preventable C-sections in India after accounting for most of the medical indications over and above the pregnancies requiring C-sections as per Robson’s criteria [16,17].

Employing the socio-ecological model, it may be argued that individual, household level, community, healthcare, and societal factors influence a pregnant women’s probability of undergoing a preventable C-section. Earlier studies reported that at an individual level, the age of the mother, health status, gravida, and education level influenced CS rates [18,19]. At a household level, specific factors such as household wealth, religion, caste, and family size were found to bee associated with CS deliveries [20]. Other healthcare and community factors such as the number of ANC visits, place of delivery, birthweight of the child, urban & rural differentials, access to health insurance, etc., were also found to be associated with CS deliveries in India [21,22].

As per the literature search, most studies included only CS deliveries, which may or may not be medically warranted. Limited literature quantifies the prevalence of preventable CS and its associated factors. Preventable C-sections result in inefficient use of health system resources, intra-operative risk, and post-operative complications that impact the mother’s and child’s future health and well-being. Available studies also show that preventable CS can result in high out-of-pocket expenses and debt and can push families into poverty [23,24]. References pointing to the longer-term impact of C-sections on mothers are also available [25–27]. The alarming rise in C-sections is a public health problem that needs to be prioritized at the highest level. Identifying preventable CS can help rationalize CS deliveries, thereby facilitating the optimization of resources and improving the health and well-being of the mothers. Moreover, the findings can facilitate the setting up of actionable targets to reduce the number of preventable CS. This study analyzed the data of the National Family and Health Survey-5 to address the following objectives: firstly, to estimate the prevalence of preventable c-section deliveries among women aged 15–44 years residing in select Indian states (Tamil Nadu, Andhra Pradesh, Telangana, Karnataka and Kerala) and secondly to examine the influence of socio-demographic, economic, health system, community and individual factors on the odds of preventable c-section deliveries among women aged 15–44 years in these Indian states. The results of the study will help policymakers and program managers delve deeper into this issue of unnecessary/ preventable caesareans in states that have good obstetric facilities and standard public health systems.

## Methods

### Data source

The study used the micro-level data of the women surveyed in the fifth round of the National Family Health Survey (NFHS–5) carried out during 2019–2021. The data was obtained from the Demographic and Health Survey (DHS) website. NFHS employs standardised processes and tools for sample identification and data collection. The NFHS protocol and tools were approved by institutional review boards of the International Institute of Population Sciences (IIPS). The NFHS protocol, survey tools, process of sample identification, and data collection are available in the public domain and were reported in the latest NFHS-5 report [6].

**Fig 1:**
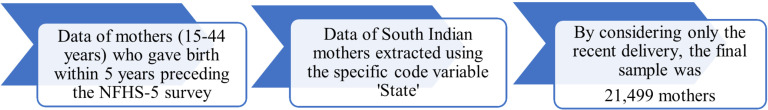
Sample size of the study.

Data for the study were extracted from the women’s questionnaire of the NFHS-5 survey, focusing on women aged 15–49 who gave birth within the last five years. The sample included 21,499 women from South Indian states—Tamil Nadu, Andhra Pradesh, Telangana, Karnataka, and Kerala, as these states have a higher percentage of institutional deliveries compared to the national average. This selection criterion ensures that the findings are reflective of regions with better healthcare access and delivery outcomes (Fig 1).

The analysis focused on each mother’s latest childbirth to reflect current delivery practices. This approach helps accurately capture the current institutional delivery practices and trends within these selected states. Caesarean sections were coded as ‘1’ *(ever had)* and ‘0’ *(never had)*. Sample weights were used in all the analysis.

### Outcome variable

The dependent variable, termed *‘Preventable C-section delivery’* was defined based on an operational framework derived from Robson’s criteria [16,17,28,29], which identifies caesarean deliveries that could have been avoided under ideal circumstances. The *Preventable C-section deliveries* is defined as a full-term caesarean performed after labor onset, without complications like prolonged labor, breech presentation [16,28], or excessive bleeding [21]. The classification was based on gestational age, mode of delivery, timing of the decision, and absence of intrapartum complications [30].

The criteria were used to compute a binary variable, categorizing caesarean sections as *‘Yes’ (Preventable)* or *‘No’ (Non-preventable)*. Detailed criteria and methods for defining and coding both the dependent and independent variables are outlined in S1 Table, ensuring a clear understanding of the Robson’s classification process.

### Other variables of the study

Considering the available literature on caesarean deliveries, we took the following variables for our study ‘Age- 15-24y, 25-34y, 35-44y; Caste- SC, ST, OBC, Others; Education- No Schooling, Primary level schooling, Secondary level schooling, Higher level schooling; Wealth Index- Poorest, Poorer, Middle, Richer, Richest; Area of residence- Urban, Rural; Family Size- Nuclear Family and Joint Family; Health Insurance- Not insured, Insured; Parity- Primigravida (a woman who is pregnant for the first time) and Multigravida (a woman that has been pregnant for at least a second time.), ANC visits- Less than 4 ANC, 4 or more ANC. Place of Delivery- Public, Private health facility; Birth Weight of the infant- Less than 2.5 Kg, 2.5 Kg to 3 Kg, 3 Kg to 3.5 Kg, above 3.5 Kg and Female Sterilization- Yes, No.

### Statistical analysis

The data analysis was conducted using Stata, version 16. The weighted prevalence rates for C-section deliveries and preventable C-sections are calculated with a 95% confidence interval. Bivariate analysis employing a chi-square test was conducted to identify factors associated with Preventable C-section deliveries across the states studied. The correlates for C-section deliveries are identified based on the socio-ecological framework to explain health behaviours.

The multivariate analysis employing binary logistic regression was conducted to identify the predictors of Preventable C-section deliveries. The variable *‘Preventable C-section delivery’* was the binary dependent variable, and the variables identified using bivariate analysis were the independent variables for the logistic regression analysis. Multi-collinearity was assessed using the variance inflation factor. Adjusted odds ratios and 95% CI were computed for both CS and Preventable CS.

### Ethics approval

The data used in the study are available in the public domain, and no identifiable information on the survey participants is provided; therefore, no ethics statement is required by us for our current study. The National Family Health Survey (NFHS) is one of the largest health surveys in the world, conducted by the International Institute for Population Sciences (IIPS), Mumbai, under the Ministry of Health and Family Welfare, Government of India.

## Results

The study sample included 2,1499 mothers aged 15–44 from five Indian states. The sample characteristics of the study population can be seen in Table 1. The maximum number of females were from the age group 25–34, 61%. Across all the states, 57% of women were secondary educated. Around 60% of the women belong to rural areas and around 40% to urban areas. Around 38% women were sterilized after giving birth.

**Table 1:** Sample characteristics of the study population, 2019–21.

Variables	States					
	Tamil Nadu	Andhra Pradesh	Telangana	Karnataka	Kerala	Total (%)
**Age category** (Completed years)						
15–24	27.5	41.6	35.8	30.7	20.1	31.0
25–34	64.7	53.0	59.5	61.2	61.8	60.6
35–44	7.8	5.4	4.7	8.1	18.2	8.4
**Caste**						
SC	28.6	25.0	22.5	20.2	10.3	22.5
ST	1.9	5.4	8.3	11.7	1.5	5.9
OBC	67.5	50.3	59.6	54.8	61.2	59.2
Others	2.0	19.3	9.6	13.2	27.0	12.4
**Education**						
No schooling	1.5	12.1	13.9	9.7	0.1	7.0
Primary level	4.5	10.9	5.9	7.0	0.4	5.9
Secondary level	53.0	58.1	55.6	64.8	53.3	57.4
Higher level	41.0	18.9	24.6	18.5	46.3	29.7
**Wealth Index**						
Poorest	2.6	4.5	3.9	6.7	0.8	3.9
Poorer	12.0	19.3	15.4	18.2	3.1	14.2
Middle	28.1	32.3	28.7	28.9	16.3	27.6
Richer	33.0	28.2	29.5	28.2	38.8	31.2
Richest	24.3	15.6	22.5	18.0	41.0	23.1
**Area of Residence**						
Urban	45.6	29.5	39.0	38.8	47.8	40.4
Rural	54.4	70.5	61.0	61.2	52.2	59.6
**Family Size**						
Nuclear family	85.8	82.0	79.6	64.3	76.3	77.4
Joint family	14.2	18.0	20.4	35.7	23.7	22.6
**Health Insurance**						
Not insured	75.7	43.3	55.6	82.4	55.0	66.3
Insured	24.3	56.7	44.4	17.6	45.0	33.7
**Parity**						
Primigravida	40.9	32.4	32.5	37.3	38.6	37.1
Multigravida	59.1	67.6	67.5	62.7	61.4	62.9
**ANC visits**						
Less than 4 ANC visits	8.9	31.7	29.4	28.4	9.2	20.8
4 or more ANC visits	91.1	68.3	70.6	71.6	90.8	79.2
**Place of Delivery**						
Private Sector	34.2	47.7	48.1	34.5	65.6	42.7
Public Health Facility	65.8	52.3	51.9	65.5	34.4	57.3
**Birth weight of Infant**						
Less than 2.5 Kg	26.4	37.6	33.8	34.5	20.8	30.7
2.5 Kg to less than 3 Kg	42.0	40.2	44.5	38.6	42.0	41.1
3 Kg to less than 3.5 Kg	24.1	16.9	16.5	19.9	28.5	21.3
3.5 Kg and above	7.6	5.3	5.2	7.1	8.7	6.9
**Female Sterilization**						
No	63.6	49.9	59.8	64.0	72.1	61.9
Yes	36.4	50.1	40.2	36.0	27.9	38.1

Note- ANC- Antenatal Care, OBC- Other Backward Castes, SC: Scheduled Caste, ST- Scheduled Tribes.

### Caesarean section and preventable caesarean section deliveries

The prevalence of ‘preventable caesarean section’, ‘caesarean section’ deliveries and the percentage of ‘preventable CS among overall CS’ for select Indian states which are Andhra Pradesh, Kerala, Karnataka, Telangana and Tamil Nadu can be seen in Table 2. Among the total study sample of 21499, the percentage of preventable CS was 6.2% (95% CI = 5.9–6.5) and the percentage of CS was 44.3% (95% CI = 43.7–44.8). The percentage of preventable CS among overall CS was 13.9% (95% CI = 13.3–14.5). State-specific differences in CS and Preventable CS deliveries were taken using the analysis.

**Table 2:** State-specific differences in CS and Preventable CS deliveries, 2019–21.

South Indian States	Preventable CS (95% CI)	CS (95% CI)	Preventable CS among overall CS (95% CI)
Tamil Nadu	7.0 (6.5–7.6)	47.1 (46.1–48.2)	14.9 (13.9–16.1)
Andhra Pradesh	5.4 (4.8–6.0)	45.2 (43.9–46.5)	12.0 (10.8–13.3)
Telangana	8.4 (7.5–9.3)	63.7 (62.2–65.2)	13.2 (11.9–14.6)
Karnataka	6.2 (5.7–6.7)	34.2 (33.2–35.2)	18.1 (16.7–19.5)
Kerala	3.1 (2.6–3.6)	39.2 (37.2–40.7)	7.9 (6.6–9.3)
**Study Sample**	**6.2 (5.9-6.5)**	**44.3 (43.7-44.8)**	**13.9 (13.3-14.5)**

Note- CI: Confidence Interval.

### Characteristics of C-section deliveries

**Fig 2:**
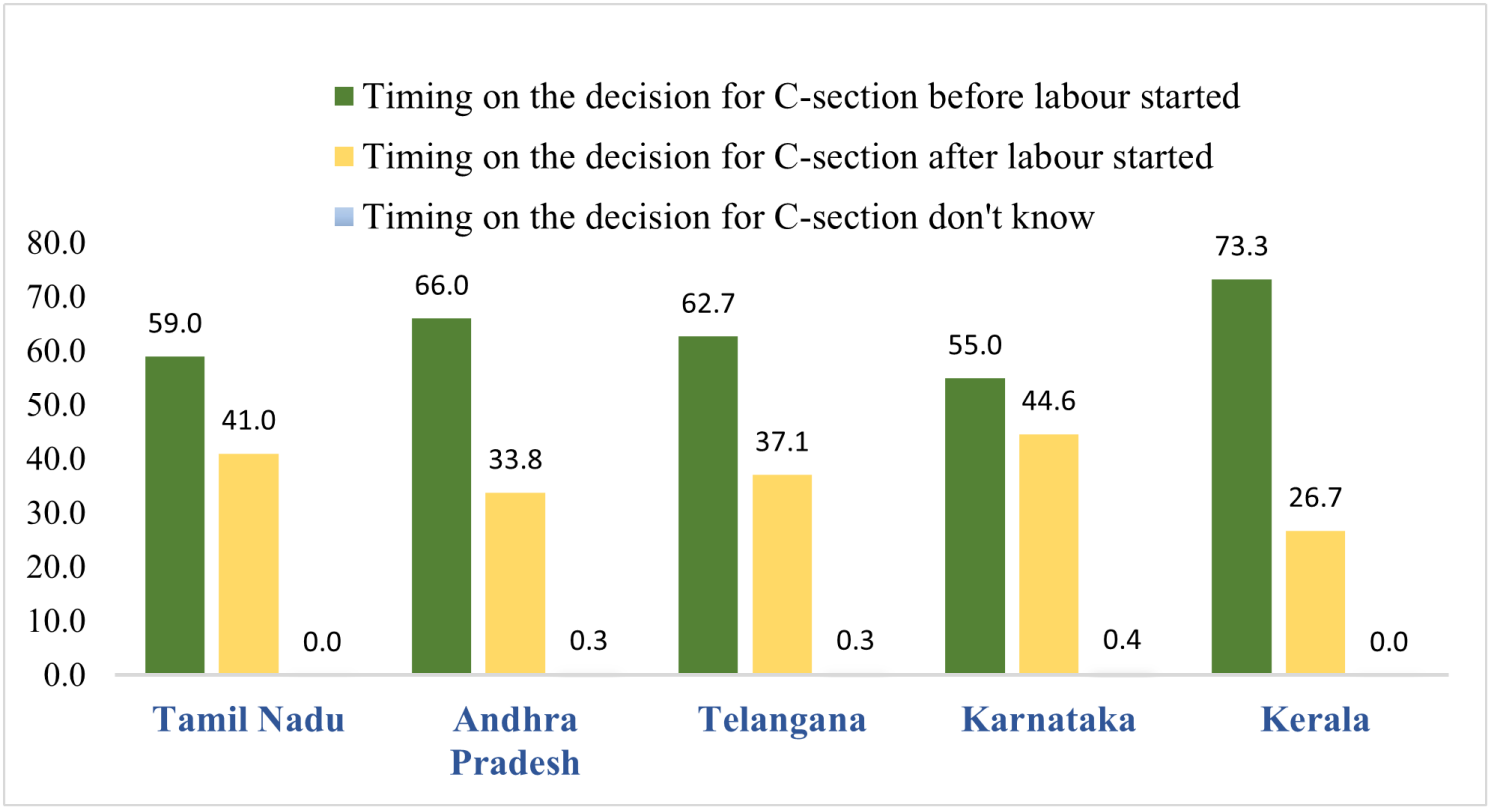
Timing of decision of CS across select Indian states, 2019-21.

The state-wise differences in the percentage of C-sections with C-section decision planned before and after the onset of labor. In Kerala, the CS planned prior to the onset of labor was higher (73.26%) compared to other south Indian states as shown in Fig 2. In Karnataka, around 45% of the CS deliveries were decided after the onset of labour.

**Fig 3:**
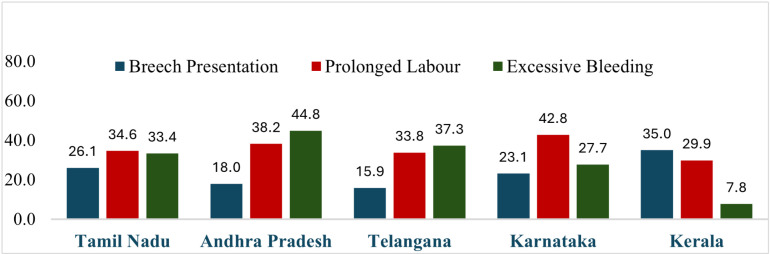
Delivery complications across South Indian states, 2019-21.

The distribution of delivery complications among C-section deliveries across select Indian states is shown in Fig 3. The delivery complications such as breech presentation, prolonged labor and excessive bleeding are shown in the figure for every state. The breech presentation is the most common delivery complication among C-section deliveries in Kerala (34.97%). Prolonged labor and excessive bleeding were the most common complications in other states.

The characteristics of the study sample with respect to CS and preventable CS is shown in Table 3. The proportion of CS and Preventable CS is higher in the 25- to 34-year-old and rural populations. Higher prevalence was found among uninsured, richer, and unemployed women, and higher Preventable CS was found to occur with increased ANC visits and in Private health care facilities.

**Table 3:** Prevalence of caesarean and preventable caesarean deliveries across various background characteristics, 2019–21.

Variables	Caesarean Deliveries (Percentage)	Preventable Caesarean Deliveries (Percentage)
**Age Group (Completed years)**		
15–24	12.2	1.9
25–34	28.3	3.8
35–44	4.5	0.6
**Caste**		
SC	9.5	1.1
ST	2.1	0.4
OBC	27.9	4.0
Others	5.6	0.7
**Education**		
No schooling	2.1	0.2
Primary level	2.2	0.3
Secondary level	24.7	3.3
Higher level	16.0	2.4
**Employment Status**		
Unemployed	35.1	4.6
Employed	9.8	1.2
**Wealth Index**		
Poorest	1.0	0.1
Poorer	4.9	0.7
Middle	12.2	1.7
Richer	14.7	2.0
Richest	12.1	1.7
**Area of Residence**		
Urban	19.4	2.9
Rural	25.7	3.4
**Family Size**		
Nuclear family	36.5	5.0
Joint family	8.5	1.2
**Health Insurance**		
Not insured	29.2	4.2
Insured	15.8	2.1
**Parity**		
Primigravida	18.0	3.1
Multigravida	27.0	3.1
**ANC visits**		
Less than 4 ANC visits	8.8	1.2
4 or more ANC visits	36.2	5.1
**Place of Delivery**		
Private Sector	25.7	3.6
Public Health Facility	19.4	2.6
**Birth weight of Infant**		
Less than 2.5 Kg	13.6	1.9
2.5 Kg to less than 3 Kg	18.2	2.4
3 Kg to less than 3.5 Kg	9.8	1.5
3.5 Kg and above	3.4	0.5
**Female Sterilization**		
No	26.1	4.0
Yes	18.9	2.2

Note: ANC – Antenatal care, OBC– Other Backward Castes, SC– Scheduled Caste, ST– Scheduled Tribe.

The results of the multivariate analysis are given in Table 4. The adjusted odds ratio (AOR) for the Preventable CS and Preventable CS among overall CS is calculated using binary logistic regression. The AOR of Preventable CS is found to be high for a higher level of education, middle socio-economic status, and with female sterilization. The AOR of Preventable CS among overall CS is high in the OBC category, the urban area of residence, and among women having sterilization. As compared to SC, the women from the OBC category were more likely to go for preventable CS deliveries.

**Table 4:** Factors associated with preventable caesarean section deliveries, 2019–21.

Variables	Preventable Caesarean Section AOR (95% CI)	Preventable CS among overall CS AOR (95% CI)
**Age Group**		
35–44 *(Ref)*		
15–24	0.66 (0.60–0.73) ^**^	1.11 (0.91–1.34)
25–34	0.80 (0.73–0.88) ^**^	1.07 (0.90–1.28)
**Caste**		
SC *(Ref)*		
ST	0.81 (0.72–0.92) ^**^	0.92 (0.77–1.12)
OBC	0.98 (0.92–1.04)	1.46 (1.13–1.89) ^**^
Others	0.82 (0.74–0.89) ^**^	1.14 (0.98–1.34)
**Education**		
No schooling *(Ref)*		
Primary level	1.32 (1.14–1.52) ^**^	0.72 (0.55–0.96)^**^
Secondary level	1.47 (1.32–1.65) ^**^	1.10 (0.87–1.40)
Higher level	1.59 (1.40–1.79) ^**^	0.90 (0.80–1.01)
**Wealth Index**		
Poorest *(Ref)*		
Poorer	1.28 (1.10–1.50) ^**^	1.05 (0.73–1.51)
Middle	1.64 (1.41–1.90) ^**^	1.11 (0.92–1.35)
Richer	1.57 (1.35–1.83) ^**^	1.07 (0.93–1.24)
Richest	1.53 (1.30–1.80) ^**^	0.97 (0.85–1.11)
**Area of Residence**		
Rural *(Ref)*		
Urban	1.01 (0.95–1.07)	1.14 (1.03–1.26)^**^
**Family Size**		
Joint family *(Ref)*		
Nuclear family	1.34 (1.26–1.42) ^**^	0.92 (0.81–1.04)
**Health Insurance**		
Insured *(Ref)*		
Not insured	0.95 (0.90–1.00)	1.07 (0.97–1.20)
**Parity**		
Multigravida *(Ref)*		
Primigravida	1.80 (1.68–1.93) ^**^	1.58 (1.43–1.78)
**ANC visits**		
4 or more ANC visits *(Ref)*		
Less than 4 ANC visits	0.97 (0.91–1.03)	0.95 (0.84–1.08)
**Place of Delivery**		
Public Health Facility *(Ref)*		
Private Sector	2.88 (2.73–3.04) ^**^	1.04 (0.94–1.15)
**Birth weight of Infant**		
Less than 2.5 Kg *(Ref)*		
2.5 Kg to less than 3 Kg	0.92 (0.87–0.98) ^**^	0.88 (0.73–1.08)
3 Kg to less than 3.5 Kg	0.92 (0.86–0.99) ^*^	0.84 (0.69–1.01)
3.5 Kg and above	1.16 (1.04–1.28) ^**^	1.01 (0.83–1.24)
**Female Sterilization**		
No *(Ref)*		
Yes	2.07 (1.94–2.21) ^**^	1.33 (1.20–1.48) ^**^

Note: ANC – Antenatal care, AOR- Adjusted Odds Ratio, CI– Confidence Interval, *Ref* – Reference, OBC– *Other Backward Castes*, SC– *Scheduled Caste*, ST– *Scheduled Tribe* and

*p < 0.05;

**p < 0.005.

## Discussion

This study examined the prevalence of preventable C-sections and identified associated factors among women who had a recent live birth within the five years preceding the NFHS-5 survey. In selected Indian states taken in the study, the prevalence of C-sections was found to be 44.3% (95% CI: 43.7–44.8), with significant variations between states ranging from 34.2% to 63.7%. These findings are comparable to those of other developing countries like Brazil and China, where C-section rates exceed 60% [1]. Several other south-Asian countries reported similar higher rates [31–33]. The 2018 Lancet series on optimizing C-sections [34] highlights that geographical disparities in socioeconomic development, female education levels, physician density, and urbanization contribute to high C-section rates, as also observed in the current study. Additionally, regional-specific factors such as inadequate public health infrastructure, widespread availability of private healthcare, and extensive health insurance coverage for C-sections, particularly in states like Telangana, may also drive the high rates of C-section deliveries [2,35].

A decomposition of C-section deliveries in a study reveals that more than 73% of C-sections in Kerala were planned before the onset of labor, and breech presentation was the most common complication reported among CS mothers [3]. While a high percentage of decisions on C-sections before the onset of labor indicates access to high-quality ANC care (as inferred from the ANC numbers of Kerala). It also reflects the possibility that a significant proportion of mothers might be opting for an elective C-section. On the contrary, excessive bleeding was a prominent complication observed in Andhra Pradesh and Telangana. Evidence from a recent meta-analysis indicates that the risk of post-partum haemorrhage is strongly associated with the absence of antenatal care [4]. It was also noted that Andhra Pradesh & Telangana were lowest regarding full ANC coverage (i.e., at least 4 ANC visits) among the states studied.

### Preventable C-sections

Our study estimates that preventable C-sections constitute between 3.1% and 8.4% of all deliveries in select Indian states. These findings align with previous research using nationally representative data, which estimated that the number of avoidable C-sections in India is at least 1.8 million [3,5]. The high prevalence of preventable C-sections in Indian states represents a significant public health challenge that urgently demands targeted policy interventions to reduce unnecessary surgical births.

### Factors associated with preventable C-sections

The findings suggest that at an individual level, the mother’s age, education, and parity were significantly associated with preventable CS. Specifically, mothers with higher education, i.e., those with primary schooling and above, have at least a 30% greater chance of undergoing a preventable C-section compared to those without education. Studies report that women with higher education tend to opt for elective C-sections [6]. The choice of a medically unwanted c-section among educated women could be multifactorial, including but not limited to economic status, workforce participation, preference for specific delivery dates, and healthcare seeking from private providers [6,8,33]. The counter-intuitiveness of higher education is associated with preventable CS deliveries indicating a potential interaction between greater choice of medical care coupled with lack of awareness about the health risks of preventable CS among educated women.

It was noted that primi-mothers had higher odds of preventable CS. A similar higher burden of CS among primi-mothers was observed in Brazil [9,12]. These findings gain prominence as 37% of all mothers in the south Indian states were primi-mothers and at least 18% of primi-mothers had undergone CS deliveries. Given that a previous CS is an indication for CS in future pregnancies [12], special attention is required to avoid preventable CS among primi-mothers. Women in nuclear families had higher odds of C-section deliveries. A mixed methods study in China reported that in societies with small family norms, CS was perceived to be a safer option than normal delivery [14]. The findings also suggest that there is a possibility of coupling sterilization (through post-partum tubal ligation (PPTL)) and CS deliveries since those who reported having undergone sterilization had twice the odds of having a preventable CS. Evidence from other parts of the world indicates the possibility of coupling of PPTL with CS [15,16]. A study conducted in a teaching hospital in Turkey reported that around 21% of the women who delivered opted for CS and tubal sterilization [17]. A study of over three million deliveries conducted in the USA reported that individuals undergoing caesarean deliveries had greater odds of using PPTL [16]. Within the study sample, sterilization rates ranged between 27.1 to 50.1% across the states. The evidence from NFHS-4 indicates that female sterilisation rates among women aged 15–49 years is 37% in India. The high prevalence of female sterilization rates might potentially be a result of a higher prevalence of preventable CS. The dichotomy of the study’s findings where preventable CS rates are high in first-order (primi-mothers) and last-order births (potentially due to sterilization), is also supported by the findings of high CS rates as reported by the NFHS-5 India report [14].

The mothers who delivered at a private healthcare facility had 2.88 odds (i.e., 2.9 times more chance) of having a preventable CS compared to mothers who delivered in a public healthcare facility. Private facilities account for 29.6% of all institutional deliveries, 48% of all deliveries in the private sector were CS deliveries, and the average cost of delivering at a private facility is Rs. 24,663. From NFHS-4 to 5, CS deliveries in private hospitals increased by 17% [18]. A study conducted in rural Maharashtra found that mothers delivering in private facilities had 1.40 times the odds of undergoing CS compared to those undergoing deliveries in public facilities [19]. Another study based on a nationally representative sample reported CS among mothers were thrice more likely to be delivering in private facilities [20]. Several other studies reported caesarean deliveries being higher in private facilities [35–37]. Evidence indicates that compared to public facilities, CS deliveries in private facilities are more pre-planned and have fewer delivery complications [22] indicating that a significant percentage of CS deliveries in the private sector are preventable. The mothers who were insured had higher odds of CS compared to those who were not covered under health insurance. While health insurance status was not significantly associated with preventable C-sections, the finding sheds light on the potential role of health insurance coverage in increasing CS rates, however some studies have discussed hospital characteristics related to higher caesarean deliveries [38–40]. Some recent study examining CS proportions also reported that higher out-of-pocket expenses and health insurance coverage are positively associated with excess CS rates [23,24].

### Strength and limitation

The strength of this study includes a large nationally representative sample of women, the evaluation of factors leading to caesarean deliveries, which allowed for a detailed description of the research population, adjustments to the analyses, and the investigation of several possible mediators. The limitation of the study is the cross-sectional design of this study which does not necessarily infer a causal relationship between caesarean deliveries and its related co-factors.

## Conclusion

The study findings indicate that preventable CS are on an increasing trajectory across the indicators of social and economic development (such as women’s education, older age at marriage and childbirth, higher wealth quintile etc). The findings emphasise the need to develop strategies to judiciously use existing strengths (i.e., high ANC coverage, institutional deliveries) to prevent unnecessary CS. Specifically, building awareness about the pros and cons of CS deliveries, and counselling for mothers and family members are needed. A significant proportion of mothers who had preventable CS also had sterilisation. The coupling of CS & PPTL can be prevented by improving awareness and access to LARC. The role of private healthcare providers in preventable CS was increasingly obvious in this study. Given the caesarean delivery rates are much higher than that recommended by the WHO hence, periodic audits of CS deliveries in all healthcare facilities and including guidelines for CS (such as reporting the medical indications for CS) could potentially assist in reducing CS rates. Insurance coverage to CS while essential, might become a potential reason for physician-induced demand and has to be strategically dealt with. A previous CS is an indication of CS in future pregnancies. So, special attention is required to avoid preventable CS among primi-mothers by building awareness about the disadvantages of CS deliveries unless medically necessary. Also, counselling for mothers and family members is needed to prevent the occurrence of preventable CS.

## Supporting information

S1 Fig Robson’s criteria. (DOCX)

S1 Table Robson’s criteria. (DOCX)

S2 Table The following details are in the National Family Health Survey – 5 Data. (NFHS – 5).
(DOCX)
