# Supplementary material for: Preventing the preventable: Assessing the burden of incessant caesarean deliveries in select Indian states using NFHS-5
Source: PLoS One. 2025 Apr 23;20(4):e0320041. doi: 10.1371/journal.pone.0320041 (PMC12017520; doi:10.1371/journal.pone.0320041)
Supplement: S1 Fig — (DOCX) [file pone.0320041.s001.docx]

**S1 fig. Robson’s Criteria**


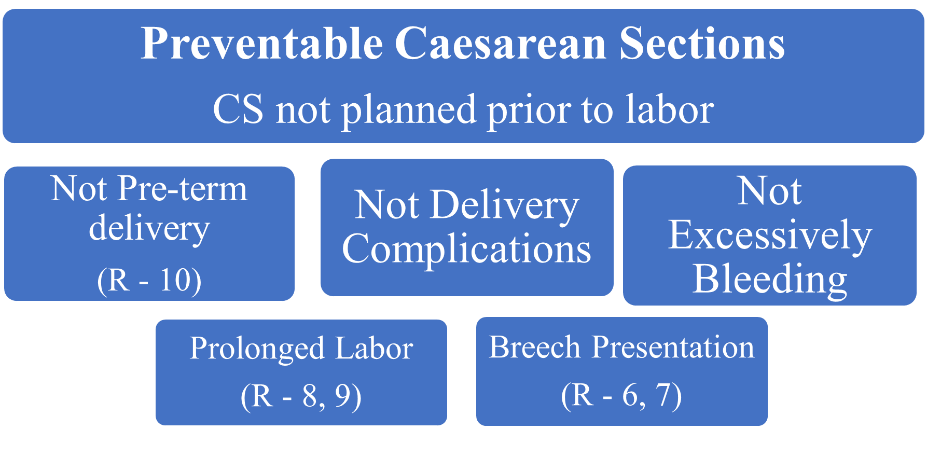


**High-risk pregnancies** are categorized from criteria 6 to 10 based on Robson’s classification. In addition, the conditions that cause the decision to elective CS based on antenatal complications and excessive bleeding are also addressed, which ensures none of the reasons for CS are missing out.
