# Supplementary material for: Preventing the preventable: Assessing the burden of incessant caesarean deliveries in select Indian states using NFHS-5
Source: PLoS One. 2025 Apr 23;20(4):e0320041. doi: 10.1371/journal.pone.0320041 (PMC12017520; doi:10.1371/journal.pone.0320041)
Supplement: S1 Table — (DOCX) [file pone.0320041.s002.docx]

**S1 Table. Robson’s Criteria**

| **Criteria** | **Shreds of evidence** |
| --- | --- |
| 1. The decision for a caesarean section (CS) was not taken prior to the onset of labor | The CS is planned prior to the onset of labor (Elective CS), in case there are any complications during the ante-natal period and has been captured in criteria 1.  The selected South Indian states have more than 95% of Institutional deliveries with proper health care referral systems and high ante-natal care (ANC) coverage, which shows a higher Continuum of care (CoC) and health system readiness. So, this would address most of the high-risk pregnancy criteria of mothers listed by the Government of India.  The improved CoC in Maternal and Child Health (MCH) will rule out the high risks such as the age of the mother (young primigravida or elderly gravida), short stature women (less than 150cm of height), HIV positive, Rh negative, Severe anemia (Hemoglobin less than 7gm/dl), placenta previa, Gestational Diabetes, Pre-eclampsia (Pregnancy-induced hypertension, Proteinuria, swelling of the body, face and feet, Convulsions without fever, headache and blurring of vision).  But these complications are **not captured by Robson’s criteria**, and it adds a greater impact on the study. |
| 1. Not a pre-term delivery | Any CS that occurred in less than 9 months is considered to be medically needed as there would be an emergency condition. And it is classified as **group 10 in Robson’s criteria** [1].  In the study, the preventable CS are captured in Full term pregnancy >37 weeks or above nine months of gestation. |
| 1. Not have experienced delivery complications of a) Prolonged labor and b) Breech presentation | **Robson’s criteria 6 and 7** address the breech presentation in nulliparous and multiparous women respectively.  **Robson’s criteria 8 and 9** address the Prolonged labor that occurs by multiple pregnancies and malpresentation irrespective of having previous CS.  Therefore, criteria 6 to 9 are acceptable CS as they are medically justified [2, 3]. |
| 1. Not had excessive bleeding during delivery. | **Robson’s criteria 5** addresses the previous CS, single cephalic full-term pregnancies.  The previous CS deliveries have higher chances of anemia, uterine rupture and excessive bleeding. And maternal hemorrhage needs blood transfusion to save the life of the mother.  Criteria 4 is not as such captured by Robson’s 5^th^ criteria. It also captures all the other possible complications which need medical care.  Literature suggests the success rate of vaginal births after CS (VBAC) is 66% [3]. So, the number of trials of labor after caesarean section (TOLAC) can be increased [4]. |
| 1. Safe or Low-risk Pregnancy Group | **Robson’s criteria 1, 2, 3 and 4**  The CS occurring in these groups are without any medical need and hence are considered preventable caesarean section.  But these safe or low-risk groups contribute majorly to caesarean section [1,2] |

**References**

1. Abubeker FA, Gashawbeza B, Gebre TM, Wondafrash M, Teklu AM, Degu D, et al. Analysis of cesarean section rates using Robson ten group classification system in a tertiary teaching hospital, Addis Ababa, Ethiopia: a cross-sectional study. BMC Pregnancy Childbirth. 2020;20(1).

2. Triunfo S, Ferrazzani S, Lanzone A, Scambia G. Identification of obstetric targets for reducing cesarean section rate using the Robson Ten Group Classification in a tertiary level hospital. European Journal of Obstetrics and Gynecology and Reproductive Biology. 2015;189.

3. Robson MS. Can we reduce the caesarean section rate? Best Pract Res Clin Obstet Gynaecol. 2001;15(1).

4. Sabol B, Denman MA, Guise JM. Vaginal Birth after Cesarean: An Effective Method to Reduce Cesarean. Clin Obstet Gynecol. 2015;58(2).
