# Supplementary material for: Preventing the preventable: Assessing the burden of incessant caesarean deliveries in select Indian states using NFHS-5
Source: PLoS One. 2025 Apr 23;20(4):e0320041. doi: 10.1371/journal.pone.0320041 (PMC12017520; doi:10.1371/journal.pone.0320041)
Supplement: S2 Table — (NFHS – 5). (DOCX) [file pone.0320041.s003.docx]

**S2 Table.** The following details are in the National Family Health Survey – 5 Data. (NFHS – 5)

| **Women’s Questions for formulating Preventable CS** | **Variable Number** |
| --- | --- |
| Delivery by C-section | M17 |
| Last birth a caesarean section | M401 |
| Duration of Pregnancy (Pre-term, Full-term or Post-term) | S220A |
| Number of times the respondent has ever given birth | V201 |
| Place of delivery (Home delivery/ Private/ Public health facility) | M15$1 |
| Received ANC for pregnancy | S414 |
| C-section was planned before/ after the onset of labor | M17A |
| **Pre-eclampsia symptoms** |  |
| During Pregnancy, had difficulty with daylight vision? | M47 |
| During Pregnancy, had convulsions, not from fever? | S434 |
| During Pregnancy, had swelling of the legs, body or face? | S435 |
| **Delivery Complications** |  |
| Did the mother experience the complication of breech presentation during delivery? | S441 |
| Did the mother experience the complication of Prolonged Labour during delivery? | S442 |
| Did the mother experience complications of excessive bleeding during delivery? | S443 |
